# Identification of trace metals and potential anthropogenic influences on the historic New York African Burial Ground population: A pXRF technology approach

**DOI:** 10.1038/s41598-019-55125-7

**Published:** 2019-12-12

**Authors:** Carter K. Clinton, Candice M. Duncan, Richard K. Shaw, Latifa Jackson, Fatimah L. C. Jackson

**Affiliations:** 1 Department of Biology, College of Arts and Sciences, Howard University, Washington, D.C 20059 USA; 2 W. Montague Cobb Research Laboratory, College of Arts and Sciences, Howard University, Washington, D.C. 20059 USA; 3 Department of Environmental Science and Technology, College of Agriculture and Natural Resources, University of Maryland, College Park, MD 20742 USA; 4 United States Department of Agriculture-Natural Resources Conservation Service, Somerset, NJ 08873 USA; 5 Department of Pediatrics and Child Health, College of Medicine, Howard University, Washington, D.C. 20059 USA

**Keywords:** Biogeochemistry, Environmental chemistry, Geochemistry

## Abstract

The New York African Burial Ground (NYABG) is the country’s oldest and largest burial site of free and enslaved Africans. Re-discovered in 1991, this site provided evidence of the biological and cultural existence of a 17^th^ and 18^th^ Century historic population viewing their skeletal remains. However, the skeletal remains were reburied in October 2003 and are unavailable for further investigation. The analysis of grave soil samples with modern technology allows for the assessment of trace metal presence. Portable X-ray fluorescence (pXRF) spectrometry provides a semi-quantitative and non-destructive method to identify trace metals of this population and in the surrounding environment. Sixty-five NYABG soil samples were analyzed on a handheld Bruker Tracer III- SD XRF with 40 kV of voltage and a 30μA current. Presence of As, Cu, and Zn can potentially decipher the influence of the local 18^th^ Century pottery factories. Elevated levels of Sr validate the assumed heavy vegetative diets of poor and enslaved Africans of the time. Decreased levels of Ca may be due in part to the proximity of the Collect Pond, the existing water table until the early 19^th^ Century, and Manhattan’s rising sea level causing an elevated water table washing away the leached Ca from human remains. These data help us reconstruct the lives of these early Americans in what became New York City.

## Introduction

Grave soil, the decomposed substrate of living organisms, is characterized by the presence of mineral remains present in the living organism and as such is used as a resource for forensic and anthropological studies. By 1664 almost 300 enslaved Africans would call new Amsterdam their home. Over the next two centuries almost 17,000 of these enslaved people and their descendants would come to be buried in the “Negroes Burial Ground” located on the southern tip of the island of Manhattan. Human grave soil remains an understudied resource that could provide a better understanding of the lived experience of the interred human. Human remains allow researchers to investigate a population that once lived but has left behind little to no record of its existence. The rediscovered burial ground, later renamed the New York African Burial Ground (NYABG), represents a high profile, partially excavated gravesite located in lower Manhattan referred to as the most significant archaeological find of the 20^th^ Century^1^. This burial ground contains a population of free and enslaved Africans who were buried between the 1640s and the 1790s in what was then known as the New Amsterdam Colony. Currently, human remains have been investigated using genomic ancient DNA sequencing methods and assessments of microbial community. These techniques have provided novel insights into human ancestry, gender identification, and historical infectious disease prevalence^2–4^.

In this investigation, we used pXRF spectrometry to detect trace metals found in 64 human- associated burial samples and their corresponding concentrations. PXRF technology is typically used in a number of environmental capacities including archaeology field studies, art conservation, and waste management. It provides a rapid, and inexpensive way to detect elements in a short time without destructive and time-consuming acid digestion^5–7^. Additionally, studies have demonstrated the accuracy of pXRF suggesting its efficacy in trace metal detection to be consistent with chemical analyses such as ICP-MS (inductively coupled mass spectrometry) and AAS (atomic absorption spectrometry)^8–18^. This technology (pXRF) was chosen for its non-destructive nature and the finite availability of these 17^th^ and 18^th^-century grave soils samples.

**Figure 1:**
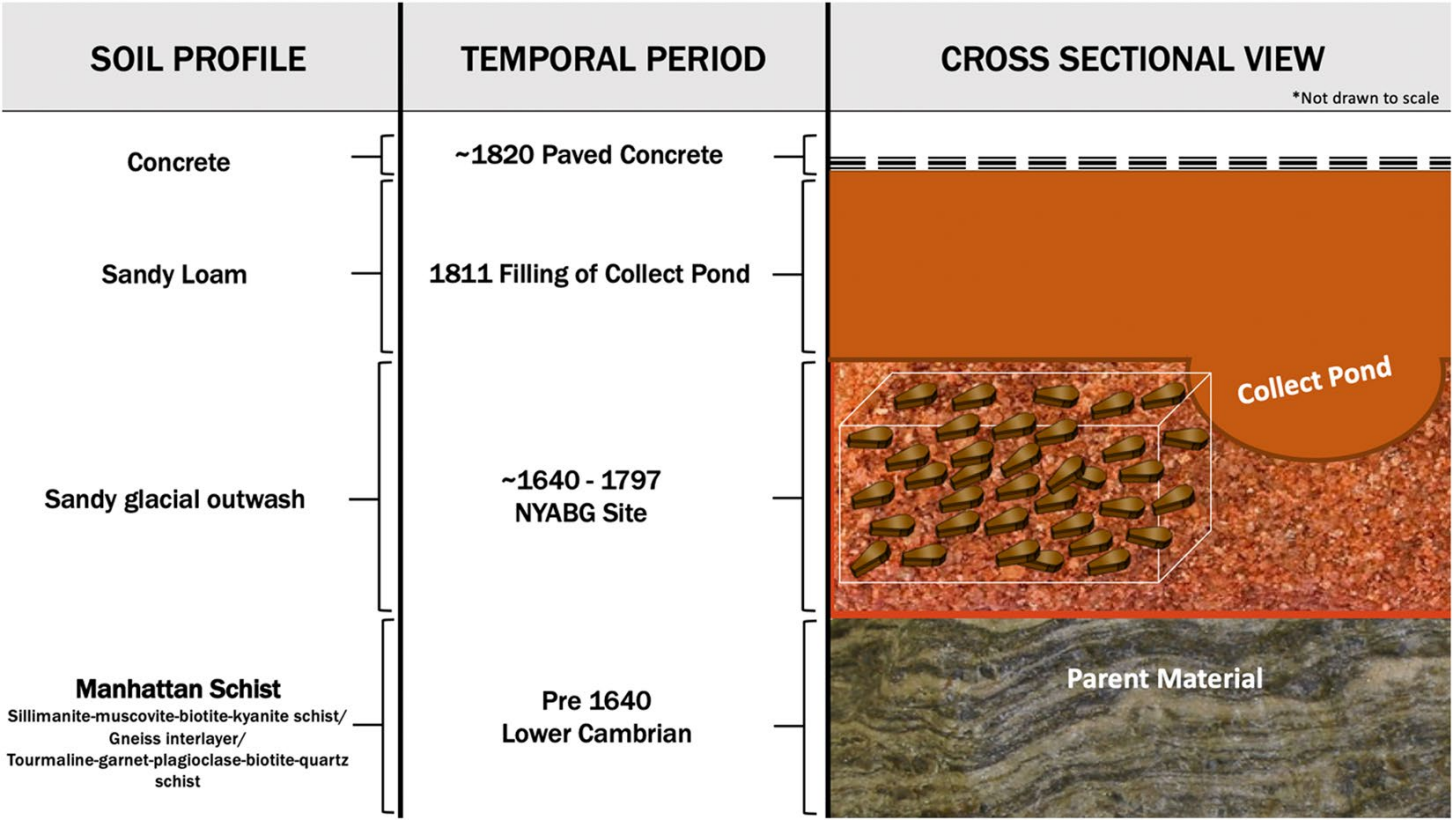
Visual representation of NYABG and control soil sample profile.

The main sources of trace elements for this investigation are decomposed human debris, their adornment and artifacts, the presence of a major water source, and multiple pottery factories. The time period for internment of NYABG inhabitants and anthropogenic contributions is ~1640 – late 1790s. Knowledge of this time period allows for determination of geologic time and subsequent soil profile based on an historical map (Fig. 1)^19^.

Previous studies have observed the impact of human remains on the presence of trace metals in soils using skeletal remains and generally using invasive/destructive analyses. As such, chemical studies are used to complement noninvasive studies using pXRF when we want to better understand the interactions between humans and their surrounding environments^20–22^. However, with pXRF technology we are able to identify elemental species and quantitatively analyze the concentrations for identification of potential factors affecting this historical population. To our knowledge, little research has explored residual trace elements strictly in grave soils, particularly 400-year old historic grave soils, to reconstruct the as-lived experiences of the population, without complete destruction of the sample.

At the time of the 1991 NYABG excavation, the skeletal and archaeological remains of 419 individuals were discovered during the initial plan to build the 30-story Ted Weiss Federal Building at 290 Broadway. The excavation site is located 2 blocks away from City Hall with Duane St. along the north border, Elk St to the east, Reade St to the south and Broadway to the west. While historical maps show the burial ground spans 6.6 acres, its vast existence was only revealed at the time of excavation. The erection of the Ted Weiss building required sub-basements that reached 30 feet below ground. Descriptions of each burial were provided in the initial reports generated shortly after the excavation by Howard University and General Services Administration (GSA). These reports detailed the skeletal biological, archaeological, and historical analyses of the NYABG^23^. The greatest depths of the coffins were recorded at 25 feet. By exploring the NYBAG soil samples we are able to extrapolate living conditions and possible contaminants of the interred individuals through trace metal analysis. Consequently, the findings of this investigation enrich the fields of archaeology, anthropology, and soil chemistry.

This paper describes a pXRF approach for the analysis of trace metals (i.e. As, Ca, Cu, Sr and Zn) in cadaveric NYABG soils and establishes a feasible protocol for similar research projects involving historic human remains. This paper also aims to describe the potential source of quantifiable trace metals in relation to anthropogenic influences during the time of internment.

## Results

Using the Bruker Tracer pXRF, we were able to detect upwards of 15 elements per sample. These elements include Fe, Cr, Pb, K, Ti, Rb, Mg, Mn, Ni, Br, Rb, Cd and the five reported in this study, As, Ca, Cu, Zn, and Sr. We only reported on the five elements that had accurate or certified values from the NYABG samples, control samples and NIST SRMs data sets. The most abundant element found in NYABG samples is Sr with an average concentration of 307.2 μg/g and the least abundant element is Ca with an average concentration of 1.9 μg/g. The average NYABG sample concentration values for the Cu, Zn, and As are 82.3 μg/g, 117.9 μg/g, and 11.5 μg/g respectively. The most abundant element found in NRCS control samples is Ca with an average concentration of 2356.4 μg/g and the least abundant is As with an average concentration value of 4.9 μg/g. The average NRCS control sample concentration values of Zn, Cu, and Sr are 41.2 μg/g, 17.8 μg/g, and 47.2 μg/g respectively (Table 1).

**Table 1:** Mean elemental concentrations of NYABG and NRCS soil samples.

Element	NYABG Mean (μg/g)	NRCS Control Mean (μg/g)
Calcium	1.9	2356.4
Copper	82.3	17.8
Zinc	117.9	41.2
Arsenic	11.5	4.9
Strontium	307.2	47.2

**Figure 2:**
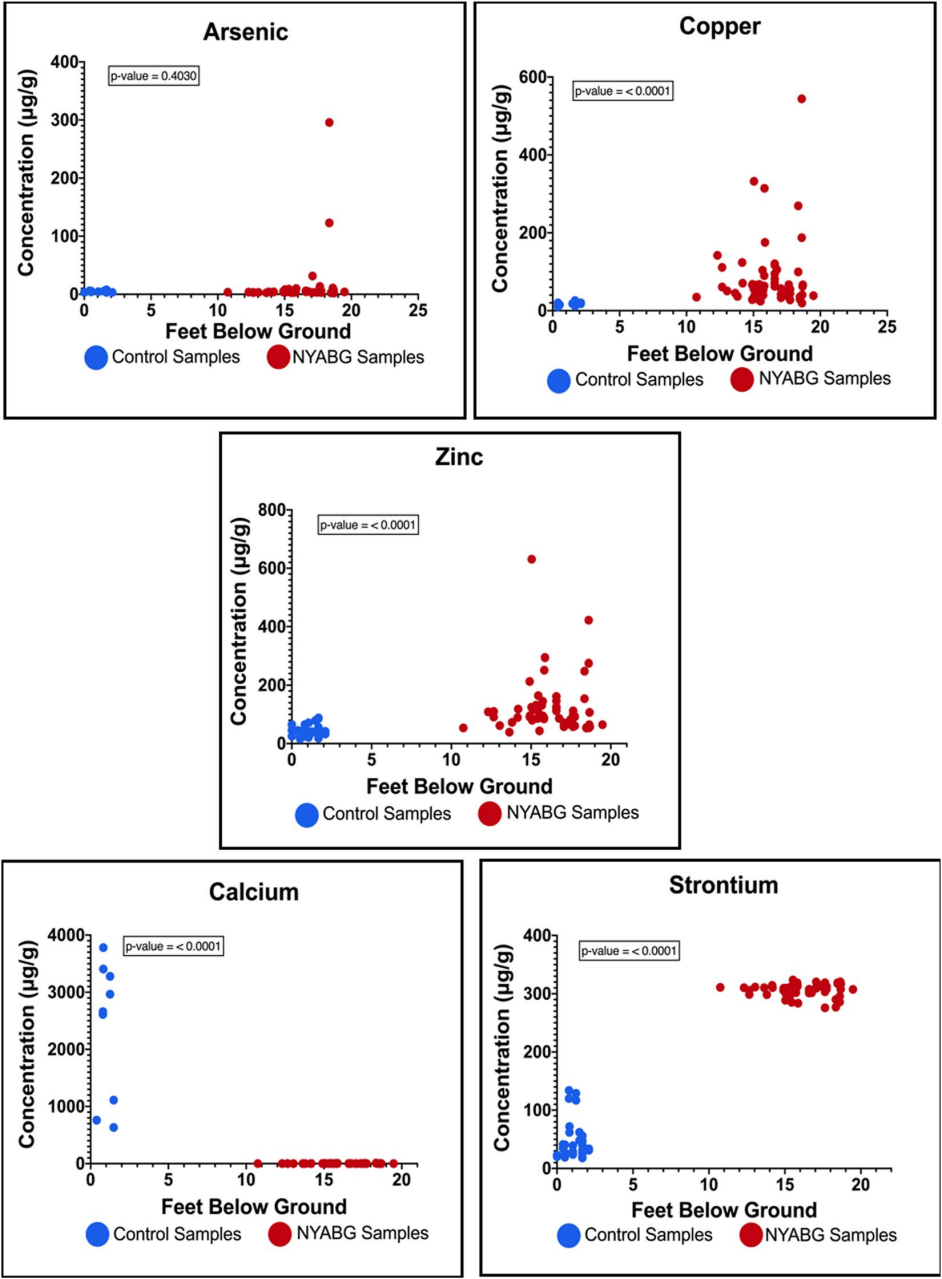
Elemental Concentration Scatter Plot of NYABG and Control samples versus depth.  Control Samples  NYABG Samples.

Comparisons between the NYABG and control samples for each element can be seen in Fig. 2. Scatterplots are shown for As, Cu, Zn, Ca, and Sr respectively. The depth for our control samples ranged from approximately 0.6 feet below ground (FBG) to 6 FBG and for the purposes of this investigation are referred to as *surface depths* as compared to our NYABG samples or *buried depths* which were found between 10 and 25 FBG. At buried depths As is present in low concentrations (<10 µg/g). At surface depths As presence is also low in concentration (i.e. <10 µg/g excluding outliers). The comparison shows no significant difference between the control and NYABG soil As samples. At buried depths Cu is present in varying concentrations from 19–544 μg/g. At surface depths Cu is present in low concentrations (<26 µg/g). The comparison shows a significant difference between the control and NYABG soil Cu samples. Similarly, a significant difference is also present in Zn, Ca and Sr as shown in Table 2.

**Table 2:** Mean elemental NYABG and control sample concentrations in correlation to depth.

Element	Buried Depths (10–25 FBG) NYABG	Surface Depths (0.6–6 FBG) Control
As	<10 μg/g	<10 μg/g
Cu	19–544 μg/g	<26 µg/g
Zn	39–631 μg/g	<88 µg/g
Ca	<11.1 μg/g	632–3780 μg/g
Sr	276–343 μg/g	18–134 μg/g

Statistical significance was determined using the Mann Whitney U- test. This method was performed using a two-tailed test to show the differences between medians of two independent samples which were not expected to display a normal distribution. An exact p-value (P < 0.0001) for Ca, Cu, Zn and Sr shows that the values of NYABG samples and values of control samples are indeed as hypothesized, significantly different. The values of NYABG samples and values of control samples of Ca, Cu, Zn and Sr are statistically significant in relation to depth. Conversely, the NYABG and control samples for As are not statistically significant in relation to depth.

**Figure 3:**
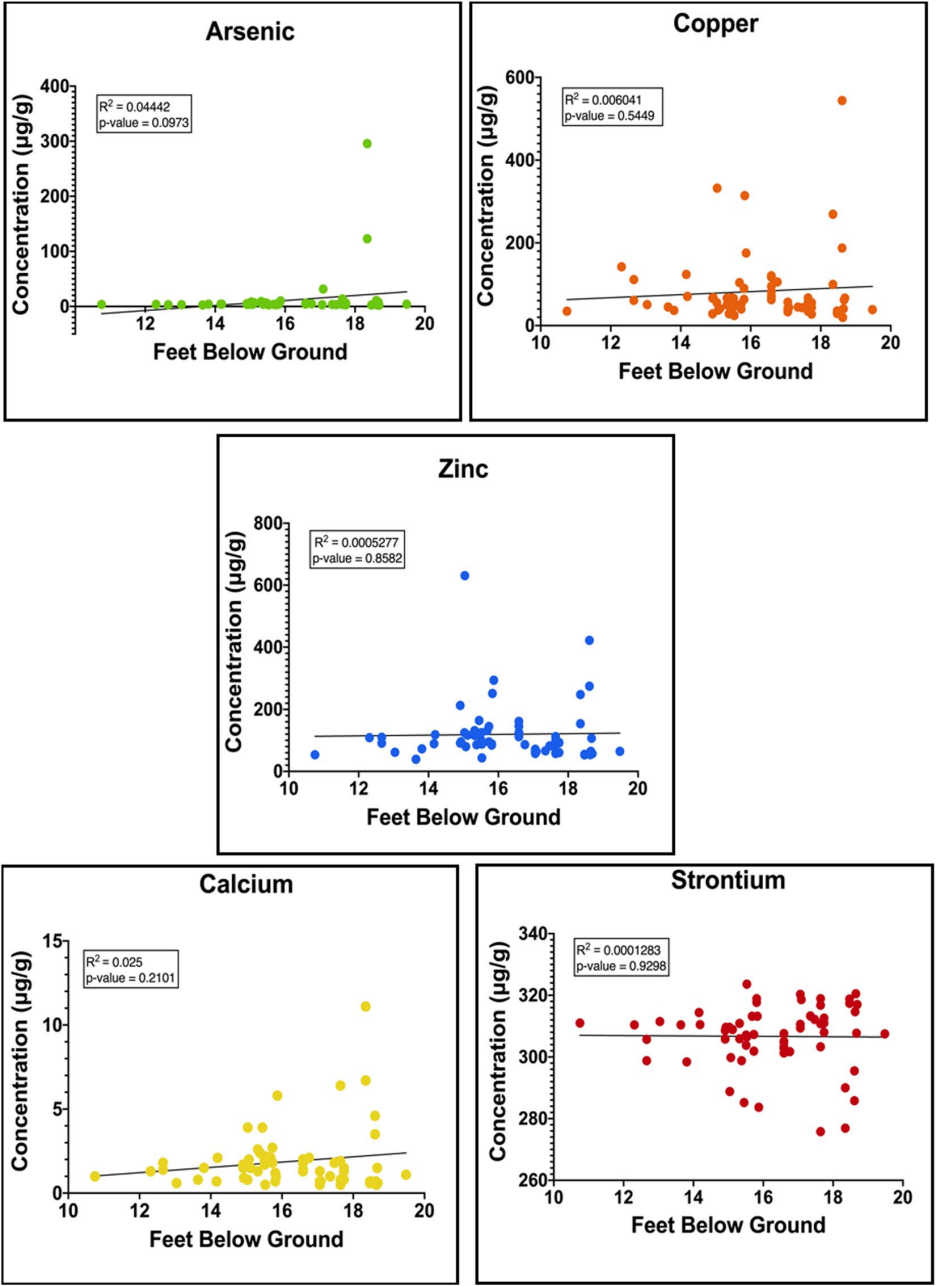
Elemental concentrations versus depth for NYABG samples with regression line.

To discern the relative elemental composition (Ca, Cu, Zn, As, and Sr) of NYABG grave soil samples generated using pXRF technology the concentration values were graphed to visualize the correlation of elemental concentrations and depth (Fig. 3). All depth information retrieved from the GSA archaeological reports^24^ were reported in average mean feet above sea level (AMSL). Statistical significance was determined using linear regression analysis displayed by the regression coefficient. All regression data show no linear correlation between element concentration and depth. P-values for each element (all p > 0.05) suggest there is no significant depth distribution of trace metals. Consequently, elemental deposits originate from the NYABG time period.

## Discussion

We use pXRF analysis to non-destructively assess the trace metal composition of cadaver-associated grave soil derived from the cranium, torso, and lower body of previously interred enslaved African Americans living in New York City during the 17^th^ and 18^th^ centuries. We found evidence of potential influences from 18^th^ Century pottery factories, the rising sea level of New York City, and the vegetative diets of the NYABG population. Previous heavy metal research on the NYABG has been conducted on the skeletal remains. The NYABG grave soil samples have never been explored. Limited studies on elemental concentrations of the skeletal remains have been performed on this population including elemental signature analysis (ESA) on enamel, dentine, and cementum of teeth using laser ablation, inductively coupled plasma- mass spectrometry (ICP-MS), and ^87^Strontium to ^86^Strontium isotopes^25^. However, NYABG grave soils have never been systematically studied for their trace metal composition, until now. This research provides new and complementary insights into the source of trace metals and their ability to serve as indicators into the lifestyles of this historical community. We contribute elemental concentration data for five elements (Ca, Cu, Zn, As, and Sr), some of which support previous ESA findings, about the NYABG population and the surrounding environment^25^.

### Arsenic, copper, and zinc

We infer that the presence of As, Cu, and Zn concentrations in the NYBAG samples reflect the anthropogenic contribution of 18th Century pottery factories, two of the first kilns built in the Colonies. This inference is based on the approximate locations of the factories and the chemical gradient of Arsenic, Copper, and Zinc that show higher concentrations closer to the pottery production sites. The Crolius Pottery factory and Corselius Pottery stoneware factory were opened in 1730 and 1735 respectively. The Corselius factory later came to be known as the Remmey and Crolius Pottery factory in 1742^26^. These pottery factories along with a potash factory were located just north of the northern border of the excavation site along today’s Duane St.^27^. They regularly used the site as a dumping ground for their kiln waste as they saw this area as “undesirable land”^1^. We found burials with the highest concentrations of As, Cu, and Zn in the northern section of the excavation site in close proximity to Duane St. In a 1998 study by Hirtle *et al*., of kiln emissions measured at 50 sites, the presence of As, Cu, and Zn were always present^28^. Studies show that As, Cu, and Zn are among the most common heavy metals found in contaminated soil at industrial waste sites. These metals can be detected years after they’ve been introduced into the environment since soil serves as a reservoir and they cannot be biodegraded^29–31^. In the case of Cu, we see increased concentrations varying throughout the burial ground. In few burials we do not see increased concentrations of As or Zn, particularly in burials where copper artifacts or adornments were reported in the initial reports^24^. Therefore, we posit the existence of these elements is evidence of kiln waste and burial artifacts including copper rings, buttons, and decorations of interred individuals deposited during the and 17^th^ and 18^th^ Centuries.

### Calcium

The abundance and availability of Ca, unlike the other metals analyzed in this investigation, is highly dependent upon other geochemical processes occurring in soil. More frequently, Ca is present in its soluble form and adsorbed to the soil complex as a positively charged cation^32^. Additionally, Ca and Mg cations leach most frequently in soils leading to a decreased concentration at varying depths^33^. The factors affecting the availability of Ca in soil are pH, cation exchange capacity (CEC), cation competition, sodium content, and sub-soil/ parent material. Excess sodium (Na) competes with Ca and other cations in areas composed of shale or sandstone, the soil type described in the initial reports of the NYBAG^24^, resulting in lower levels of Ca^32^. Calcium, the least abundant element found in NYABG samples, may exist in low quantities as a result of one or several of the previously mentioned factors. We suspect Ca leached from NYABG skeletal remains to the soil and were desorbed by the wetland environment of the New Amsterdam Colony of the 17^th^ and 18^th^ Centuries. Historical maps of NYC show the Collect Pond with streams flowing east to the East River and west to the Hudson River^34^. This body of water was New York’s main water source and responsible for much of the wetland environment until 1811 when it was filled in by leveling Bayard’s Hill^35^. By the 1990s we see a similar contribution of water beneath Lower Manhattan, a result of the rising sea level due to increasing temperatures in the atmosphere. It has been reported that one foot of sea-level rise has occurred since 1900 due in part to warming of the ocean water^36^. We suppose this rising water table may be desorbing Ca from the soil and washing it away west to the Hudson River or the East to the East River located approximately 1.5 miles away.

### Strontium

Sr is commonly used when investigating ancient populations to infer characteristics about their biological and cultural existence. Generally, in studies of human bone remains, Sr is studied using isotope analysis. This method was used to generate Sr data reported in the initial reports on the NYABG^26^. In a 2000 study by Beard and Johnson, they report Sr isotope composition measured from skeletal remains can be used to infer their geographic habitat^37^. Biogenic ^87^Sr/^86^Sr analysis of enamel, dentine, and cementum was studied to distinguish the New World Africans from the Old-World Africans buried in the NYABG. As expected, the juveniles were born in New York and the majority of adult individuals, were born in Africa^26^. Additionally, researchers used ESA to explore Sr and Ba to Ca ratios to evaluate trophic level of diets. It has been well documented that ratios of Sr and Ba in relation to Ca are indicators of meat presence in diets^26,38–44^. It is fundamentally important to understand the human and biological culture of the NYABG to archaeologists and anthropologists^45^. Under normal metabolic circumstances Sr/ Ca ratios help researchers to investigate the tendency of human populations towards meat vs. vegetable consumption^44^. Elevated Sr suggests the interred individuals of the NYABG maintained a largely vegetative diet as opposed to a meat-based diet^37–45^. This aligns with the historical assumptions about the socio-economic status of these people. As enslaved or poor free Africans in the New Amsterdam Colony, this population lacked access to robust, protein-rich foods. Consequently, they consumed only the leafy and root crops they cultivated from the surrounding land.

## Conclusion

We demonstrate the utility of pXRF technology to successfully and conservatively detect trace elements in burial soil allowing for the deduction of major events, daily routines, and cultural influences in the lives of the enslaved population of 17^th^ and 18^th^ Century New York City. PXRF technology helps us extrapolate elemental signatures of the remaining fraction of the burial ground and present-day lower Manhattan that is now covered with concrete and cannot be sampled. Our conclusions indicate heavy metal distribution in the NYABG, indicators for predominant vegetative diet in a historical population, and possible signs of local climate change. This research establishes evidence for the value of pXRF technology to reconstruct past human activities in soil. Understanding these findings is relevant for similar studies of cemetery soil investigations, ancient human populations, forensic science, and anthropology. PXRF technology provides a mechanism for discovering details of the historical presence of populations that are currently inadequately documented. Researchers can now make connections between past populations and contemporary ones. PXRF makes these discoveries possible with minimal starting material and low-cost experimentation. We intend to follow this pXRF examination of grave soil remains up with most conventional chemical analyses such as ICP-MS and ICP-OMES. These additional analytic techniques are useful in determining the speciation and source of heavy metal deposition of these elements within a sample. These destructive methods were avoided as they require consumption of limited grave soil samples of significant historical interest.

## Materials and Methods

### Study design

This investigation looks at 325 pelleted soil subsamples analyzed from the decomposed remains of 44 burials to elucidate anthropogenic and environmental conditions of the NYABG population and Lower Manhattan. These 44-cadaver associated burials resulted in the collection of 65 soil samples chosen by opportunistic sampling. The collection is on loan to the W. Montague Cobb Research Laboratory (CRL) at Howard University from the National Park Service. All burial samples containing >20 g of soil were chosen for analysis. Burial samples containing <20 g of soil were not used for pXRF analysis. We collected subsamples of each burial sample for pXRF analysis. The remaining sample will be used for future chemical analysis. The subsamples were particle size sifted using a 250 μm and formed into a cylindrical pellet with a urea binder. The pellet process creates a uniform weight, shape, and size for each sample reducing the chances of variation in element detection. Each pellet was analyzed on a non-invasive, semi-quantitative, portable Bruker Tracer SD-III XRF spectrometer^46^ to detect the wavelength of each element in each sample. Bruker Tracer S1PXRF and ARTAX^47^ software packages were used to interpret the atomic spectrum identified by the device. The output from these programs were analyzed for statistical significance and interpreted based on reports from the initial excavation, historical context, and pre-existing scientific literature.

### NYABG sample site

The NYABG site at the time of excavation is believed to be approximately 25 feet below ground surface. According to the U.S. Geological Survey New York City Folio surficial geology map, the parent material for soil formation at this location is stratified drift^48^.

### Control sample site

The control samples, from throughout New York City, represent the soils formed in stratified drift (glacial outwash) found in the city and potentially found at the site^49^. Soil material from the lower subsoil and substratum horizons, which reflect natural background levels away from any anthropogenic inputs, were analyzed.

### Samples

#### NYABG samples

The NYABG collection consists of 92 burial samples with multiple body region samples from 59 individuals. Due to low mass (i.e. less than 5 g), only 64 burial samples corresponding to 44 individuals were analyzed. At the time of excavation, each sample was carefully collected in cloth bags (later transferred to amber bottles), labeled with the burial number, catalog number, and region of the body from which it was collected. The burials were sampled from body regions ranging from the cranial pedestal, pelvis, and bones of the feet. One non-burial associated sample labeled Burial N/A was collected. A total of 65 burial samples were analyzed five times producing 325 pellets.

#### Control samples

Control samples and subsequent data was provided by the United States Department of Agriculture- Natural Resources Conservation Service (USDA- NRCS) from National Cooperative Soil Survey. The control samples were collected from 31 sites in the NYC area (Staten Island, Brooklyn, and Queens) representing 6 soil profiles.

### Reference samples (Standard Reference Materials – SRMs)

The reference samples or Standard reference materials (SRMs) were obtained from the National Institute of Standards and Technology (NIST) in Gaithersburg, Maryland for the sole purpose of creating calibration curve data. The five SRMs used to achieve calibration curve data are SRM1646a Estuarine sediment, SRM1944 New York/ New Jersey Waterway sediment, SRM 2709a San Joaquin soil, SRM2710a Montana I soil, and SRM2711a Montana II soil.

### Sample preparation

The pellet press used to conduct the research was made available via the collaboration between the CRL and the University of Maryland, College Park, Department of Environmental Science (UMD-ENST) and Technology. Initial processing, categorizing and archiving was performed at Howard University. The samples were retrieved from the artifact cabinet, weighed to calculate their starting mass and determine whether enough soil was available for the proposed analyses. They were then transferred to amber glass bottles to protect them from UV exposure, moisture, and fluctuating temperatures. The bottles were then labeled according to that which was on the cloth bags.

Ten grams of grave soil were subsampled from each of the 65 samples and sieved using a No.60 sieve (250 μm mesh). The 10 grams were divided into five 2 g samples and listed A-E. For example, Burial 202 was labeled 202A, 202B, 202C, 202D and 202E, each with a 2 g mass. The sieved soil was converted from its soil particle form to a suspended mixture using a 0.2 mg/mL concentration urea binder. The mixture was then pressed using a manual Parr pellet press^50^. This process was repeated five times, creating five 2-g pellets for each sample. The samples were left to dry between 24 and 72 hours.

### Sample analysis

The 325 pelleted samples were placed atop a stationary handheld Bruker Tracer SD-III XRF spectrometer. Each sample run for 120 seconds at a voltage of 40 kV and a 30μA current using the red filter. The red filter is designed to enhance the detection of heavy metals such as Hg, Pb, Br, and As without use of a vacuum^51,52^. The output collected from pXRF analysis generated data for approximately 15–25 elements for the range of light elements (Cu, Fe, Ca and K) to heavier metals (Ar, Pb, and Cr), and excludes the periodic table in totality. This investigation confidently reports on the findings for Cu, Ca, Sr, As, and Zn due to their overlapping availability in our burial samples, control samples, and SRMs. Of the approximate 25 detectable elements, 5 were quantifiable using the certified SRM data. All concentration values (net photon count) and spectrum output (K-alpha) were generated using the proprietary Bruker Tracer and ARTAX software. All elements of interest (As, Ca, Cu, Sr, and Zn) were found in the K-alpha series of the spectrum. Their concentrations (net photon count) were identified using a specific region of interest (ROI), a portion of the XRF spectrum representing a range of energies corresponding to a particular peak or x-ray emission line^53^. The net photon count of each sample was converted to μg/g using SRM calibration curves to quantify experimental samples. The regression coefficients (R^2^) for calibration curves were 0.997, 0.996, 0.994, 0.992, and 0.981 for copper (Cu), zinc (Zn), arsenic (As), calcium (Ca) and strontium (Sr) respectively using the certified SRM.

The average photon count of the 65 samples was generated using the five A-E sample pellets. This was performed to show uniformity through the pelleting process, observe potential variability within the samples and ensure confidence in repeatability of the analysis. Comparative statistical data was carried out using Graphpad Prism 8 software^54^.

Control samples were analyzed at USDA-NRSC in Sumerset, NJ on a Delta standard model portable x-ray fluorescence spectrometer (Olympus Innov-X, Woburn, MA, USA) with an Au/Ta x-ray tube operated at 40 kV and calibrated with a “316” stainless steel alloy. The instrument was operated in soil mode configuration with three beam sequential scanning for a total analysis time of one minute with each sample run twice and an average value reported.

## Supplementary information

Supplementary Info

## Data Availability

The New York African Burial Ground soil sample dataset belongs to the W. Montague Cobb Research Laboratory at Howard University. This dataset is not publicly available yet, since it’s actively being used for the completion of a doctoral dissertation. However, these data are available from the corresponding author for means of collaboration or by reasonable request. The United States Department of Agriculture- Natural Resources Conservation Service (USDA- NRCS) control sample data were collected from the National Cooperative Soil Survey support data of New York City. Data are available upon request to corresponding author. Details can be found at: https://www.soilandwater.nyc/uploads/7/7/6/5/7765286/reconnaissance_soil_survey_report.pdf.
