# Supplementary material for: Identification of trace metals and potential anthropogenic influences on the historic New York African Burial Ground population: A pXRF technology approach
Source: Sci Rep. 2019 Dec 12;9:18976. doi: 10.1038/s41598-019-55125-7 (PMC6908665; doi:10.1038/s41598-019-55125-7)
Supplement: Supplementary file 1 — Supplementary Info [file 41598_2019_55125_MOESM1_ESM.docx]

SUPPLEMENTARY MATERIALS

**Identification of trace metals and potential anthropogenic influences on the historic New York African Burial Ground population: A pXRF technology approach**

*Carter K. Clinton^a,b,1^, Candice M. Duncan^b,c^, Richard K. Shaw^d^, Latifa Jackson^b,e^, and Fatimah L. C. Jackson^a,b^

^a^ Department of Biology, College of Arts and Sciences, Howard University, Washington, D.C, 20059

^b^ W. Montague Cobb Research Laboratory, College of Arts and Sciences, Howard University, Washington, D.C., 20059

^c^ Department of Environmental Science and Technology, College of Agriculture and Natural Resources, University of Maryland, College Park, MD 20742

^d^ United States Department of Agriculture-Natural Resources Conservation Service, Somerset, NJ 08873

^e^ Department of Pediatrics and Child Health, College of Medicine, Howard University, Washington, D.C., 20059

T1. Control sample information provided by USDA-NRCS (line 82)

| **SITE LOCATION** | **SOIL SERIES** | **PARTICLE SIZE CLASS** | \| **LITHOLOGY** \| \| --- \| |
| --- | --- | --- | --- | --- |
| Staten Island | Branford | coarse-loamy over sandy | red sedimentary |
| Staten Island | Deerfield | sandy | granite, gneiss or schist |
| Brooklyn | Haven | coarse-loamy over sandy | granite, gneiss or schist |
| Staten Island | Penwood | sandy | red sedimentary |
| Queens | Plymouth | sandy | siliceous rocks |
| Staten Island | Windsor | sandy | granite, gneiss or schist |
